# Acetylcholinesterase Inhibitors Reduce Neuroinflammation and -Degeneration in the Cortex and Hippocampus of a Surgery Stress Rat Model

**DOI:** 10.1371/journal.pone.0062679

**Published:** 2013-05-03

**Authors:** Alexander Kalb, Clarissa von Haefen, Marco Sifringer, Annalena Tegethoff, Nadine Paeschke, Mariya Kostova, Aarne Feldheiser, Claudia D. Spies

**Affiliations:** Department of Anesthesiology and Intensive Care Medicine, Campus Charité Mitte and Campus Virchow-Klinikum, Charité, Universitätsmedizin Berlin, Germany; Universidade de São Paulo, Brazil

## Abstract

Exogenous stress like tissue damage and pathogen invasion during surgical trauma could lead to a peripheral inflammatory response and induce neuroinflammation, which can result in postoperative cognitive dysfunction (POCD). The cholinergic anti-inflammatory pathway is a neurohumoral mechanism that plays a prominent role by suppressing the inflammatory response. Treatments with acetylcholinesterase inhibitors enhance cholinergic transmission and may therefore act as a potential approach to prevent neuroinflammation. In the presence or absence of acetylcholinesterase inhibitors, adult Wistar rats underwent surgery alone or were additionally treated with lipopolysaccharide (LPS). Physostigmine, which can overcome the blood-brain barrier or neostigmine acting only peripheral, served as acetylcholinesterase inhibitors. The expression of pro- and anti-inflammatory cytokines in the cortex, hippocampus, spleen and plasma was measured after 1 h, 24 h, 3 d and 7 d using Real-Time PCR, western blot analysis or cytometric bead array (CBA). Fluoro-Jade B staining of brain slices was employed to elucidate neurodegeneration. The activity of acetylcholinesterase was estimated using a spectrofluorometric method. Surgery accompanied by LPS-treatment led to increased IL-1beta gene and protein upregulation in the cortex and hippocampus but was significantly reduced by physostigmine and neostigmine. Furthermore, surgery in combination with LPS-treatment caused increased protein expression of IL-1, TNF-alpha and IL-10 in the spleen and plasma. Physostigmine and neostigmine significantly decreased the protein expression of IL-1 and TNF-alpha. Neuronal degeneration and the activity of acetylcholinesterase were elevated after surgery with LPS-treatment and reduced by physostigmine and neostigmine. Along with LPS-treatment, acetylcholinesterase inhibitors reduce the pro-inflammatory response as well as neurodegeneration after surgery in the cortex and hippocampus. This combination may represent a tool to break the pathogenesis of POCD.

## Introduction

Peripheral inflammation in response to major surgery or infection can affect the function of the central nervous system (CNS), including memory and cognition [1], [2]. The activation of the immune system, by either lipopolysaccharide (LPS) administration or surgical trauma, has been shown to be capable of affecting hippocampal function thereby causing memory impairment [3], [4]. Postoperative cognitive dysfunction (POCD) is a threatening complication after major surgery and is independently associated with increased mortality [5]–[7]. POCD seems to be a heterogeneous and multifactorial disorder with known risk factors including advanced age, duration of surgery and postoperative infection [7]–[11]. Major surgery does appear to be one principle culprit. Yet, increased inflammatory activity triggered by surgery may play a mean role in the pathogenesis of POCD [12]–[14].

Neural reflex circuits regulate cytokine release to prevent potentially inflammation and maintain homeostasis. Sensory input elicited by infection or injury moves through the afferent vagus nerve to the brainstem. Incoming signals generate action potentials that travel via efferent nerves from the brainstem to the spleen and other organs [15]–[22]. Neurotransmitters from the peripheral autonomic nerves subsequently promote the release of acetylcholine from a subset of CD4-positive T cells which activates alpha7 nicotinic acetylcholine receptors (α7 nAChR) on macrophages [23]–[25]. Acetylcholine attenuates the production of TNF-alpha, IL-1beta, IL-6 and IL-18 by macrophages at the posttranscriptional stage [16], [26], [27]. This mechanism is called the *inflammatory reflex* and triggers the cholinergic anti-inflammatory pathway, which in turn is a physiological neuroimmune mechanism that regulates innate immune function and controls inflammation [15], [16], [21], [22]. Animal studies indicate that stimulation of the vagus nerve or administration of α7 nAChR agonists reduces pro-inflammatory cytokine production by 50–75% but does not eliminate their activity [23], [28], [29]. Target therapies that increase the activity of the *inflammatory reflex* as well as the anti-inflammatory pathway normalize innate immune responses without abolishing them or causing immunosuppression [15]. The levels of acetylcholine are continuously regulated by the hydrolytic enzyme acetylcholinesterase (AChE), which rapidly degrades acetylcholine in the periphery and the brain. AChE is expressed in cholinergic neurons and neuromuscular junctions as well as tissues that are not innervated by cholinergic neurons [30], [31]. Acetylcholinesterase inhibitors prevents the Hacetylcholinesterase enzymeH from breaking down acetylcholine, thereby increasing both, the level and action time of the Hneurotransmitter acetylcholine [32]. It has been shown that pharmacologic cholinesterase inhibition improves survival in experimental sepsis [33]. The aim of this study was to investigate if AChE inhibitors reduce stress-induced neuroinflammation by maintaining acetylcholine. Furthermore, we investigated whether there is a difference between physostigmine which can cross the blood-brain barrier and neostigmine which does not enter the CNS. Here we show how surgery alone or combined with LPS-treatment with or without application of the AChE inhibitors physostigmine and neostigmine affects the expression of pro- and anti-inflammatory genes and proteins in different brain regions, spleen and plasma of adult rats. We demonstrate that surgery accompanied by LPS-treatment enhanced pro-inflammatory cytokines in the brain, spleen and plasma, and were reduced by physostigmine and neostigmine. Additionally, we provide evidence that surgery combined with LPS-treatment triggers neurodegeneration which is also affected by physostigmine and neostigmine.

## Materials and Methods

### Animal Model

Adult male Wistar rats (age: 10 weeks, weight: 250–300 g) underwent laparotomy alone to mimic human abdominal surgery or laparotomy combined with LPS-treatment in the presence or absence of physostigmine or neostigmine. The animals were kept at room temperature (22±2°C) under standard 12–12 h light–dark cycle. Food and water were available ad libitum except for the times of experiments.

### Ethics Statement

All animal experiments were approved and performed in accordance with the guidelines of the Charité - Universitätsmedizin Berlin, Germany and the local ethics committee (Landesamt für Gesundheit und Soziales (LAGeSO), Berlin, Germany), ethical permit number: G 0253/09.

### Surgical Procedure and Drug Treatment

Rats are spontaneously breathing and deeply anesthetized using isoflurane (2,3 expirative volume % and 100% oxygen) and meloxicam (Metacam®, Boehringer Ingelheim Pharma GmbH, Ingelheim, Germany), 0,2 mg/kg body weight (BW). After midline section LPS (1 mg/kg BW) was applied into the peritoneal cave and above the omentum majus. The animals received 100 µg/kg BW of physostigmine (Anticholium®, Dr. Franz Köhler Chemie, Alsbach-Hähnlein, Germany), or the same amount of neostigmine (Neostig®, Carinopharm GmbH, Gronau, Germany), after opening the abdominal wall and five minutes before the LPS-application. After surgical treatment the animals got three times (in the morning and in the evening) the same doses of subcutaneous physostigmine or neostigmine. The vehicle-treated animals received the equivalent dose NaCl 0.9% at the same time-points. The surgical procedure was performed by the same person and lasted approximately 15 minutes and while being operated the animals lay under an infra-red heating light.

Meloxicam (0.2 mg/kg BW) was administered intramuscularly before opening the abdominal wall. This medication was given intracutaneously once daily untill the third postoperative day. Under this analgesic treatment the animals showed mild signs of distress and recovered soon.

### Tissue Sampling

After 1 h, 24 h, 3 d and 7 d the animals were sacrificed in deep isoflurane-oxygen-mixture narcosis. After thoracotomy blood was taken direct from the heart and the animals were dying via exsanguination. The organs were jetted with ice-cold phosphate-buffered saline and the brain was immediately removed. For Real-Time PCR and western blotting the spleen, hippocampal and cortical tissue of a half of the brain were snap-frozen in liquid nitrogen. The other brain hemisphere was deep frozen on dry ice for immunohistochemical methods. All samples were kept at −80°C until further processing.

### Semiquantitative Real-Time PCR

Total cellular RNA from the hippocampus, cortex and spleen was isolated from snap-frozen tissue by acidic phenol/chloroform extraction and DNase I treatment (Roche Diagnostics, Mannheim, Germany). 500 ng of RNA was reverse transcribed at 42°C (1 h) with 200 U of Moloney murine leukemia virus reverse transcriptase and 2 µM oligo d(T) 16 primer (Promega, Madison, WI, USA) in 25 µl total reaction mixture. The resulting cDNA was amplified by Real-Time PCR. The oligonucleotide primers used for the PCR reactions of *TNF-alpha*, *IL-1beta* and *IL-10* and the internal standard *hypoxanthine–guanine phosphoribosyltransferase* (*HPRT*) are summarized in table 1. The expression of the target genes and the housekeeping gene *HPRT* were analyzed by Real-Time PCR using the ABI Prism 7500 sequence detection system (Applied Biosystems, Foster City, CA, USA) according to the 2^−ΔΔCt^ method [34].

**Table 1:** Sequences of oligonucleotides and gene locus.

Gene	Oligonucleotide sequences 5‘-3‘	Gene Bank Accession No.
**IL-10** forward reverse probe	gaa gac cct ctg gat aca gct gc tgc tcc act gcc ttg ctt tt cgc tgt cat cga ttt ctc ccc tgt ga	NM_012854
**TNF-alpha** forward reverse probe	tcg agt gac aag ccc gta gc ctc agc cac tcc agc tgc tc cgt cgt agc aaa cca cca agc aga	NM_012675
**IL-1beta** forward reverse probe	aac aaa aat gcc tcg tgc tgt ct tgt tgg ctt atg ttg tgt cca ttg acc cat gtg agc tga aag ctc tcc acc	NM_031512
**HPRT** forward reverse probe	gga aag aac gtc ttg att gtt gaa cca aca ctt cga gag gtc ctt tt ctt tcc ttc gtc aag cag tac agc ccc	NM_012583

### Immunoblotting

The hippocampus, cortex and spleen were homogenized on ice in cold lysis buffer containing 10 mM Tris/HCl, pH 7.5, 300 mM NaCl, 1% Triton X-100, 2 mM MgCl_2_, 5 µM EDTA, and the protease inhibitor cocktail, Complete Mini (Roche Diagnostics). The lysates were centrifuged at 13,000 g for 15 min at 4°C to remove debris. Protein concentration was determined using the bicinchoninic acid assay from Pierce (Rockford, IL, USA). Samples with equal amounts of protein were then separated by 10% polyacrylamide gel electrophoresis, and transferred to nitrocellulose membranes. After blocking in 5% low fat milk solution, the membranes were incubated over night (4°C) with a primary polyclonal rabbit anti-rat IL-1beta antibody (0.2 µg/ml, PromoKine, Heidelberg, Germany) or monoclonal mouse anti-rat β-actin (1∶5.000, BD Biosciences, Heidelberg, Germany) in 5% low fat milk in PBST. Horseradish peroxidase-conjugated secondary antibodies (anti-rabbit and anti-mouse, Southern Biotechnology Associates, Birmingham, AL, USA) were diluted 1∶25000 in PBST. Chemiluminescent detection was performed using an ECL west pico detection kit (Pierce).

### Cytometric Bead Array

A panel of pro- and anti-inflammatory cytokines including IL-1alpha, TNF-alpha and IL-10 were simultaneously quantified by a multiplex bead array system (BD™ CBA Flex Sets, BD Biosciences) on a FACS Canto II flow cytometer (BD Biosciences). This assay provided micro-bead populations with distinct fluorescent intensities and was precoated with capture antibodies specific for the cytokines. When the beads were incubated with the corresponding phycoerythrin conjugated detection antibodies and the test sample, sandwich complexes were formed. For our analysis, 50 µl of spleen tissue homogenate, plasma or the provided standard cytokines were added to the pre-mix micro-beads and incubated for 1 h in the dark at room temperature. After addition of 50 µl detecting reagent, the mixture was incubated for two hours in the dark at room temperature. This mixture was washed and centrifuged at 200 *g* for five minutes and the pellet was resuspended in 300 µl washing buffer. The FACS Canto II flow cytometer was calibrated with the setup beads, and 1500 events were acquired for each sample. The quantities of individual cytokines as indicated by their fluorescent intensities were computed using the standard reference curve of the CBA Software (BD Biosciences).

### Immunohistochemistry

Brain sections (12 µm) were cut on a cryostat and the slices were mounted on a cover slide. The slides were incubated in a solution of 0.06% potassium permanganate for 20 min, rinsed in distilled water for 1 min, and transferred into Fluoro-Jade B (FJB) (Chemicon, Jefferson, AR, USA) staining solution (0.001% FJB/0.1% acetic acid) for 20 min. The slides were thereafter rinsed three times in distilled water and transferred into Hoechst 33342 staining solution (Sigma, Taufkirchen, Germany) for 5 min. The slides were rinsed three times in distilled water and air dried, then coverslipped with mounting media. The slides were visualized by fluorescent microscopy (Olympus, Hamburg, Germany) and digitally photographed. Because the Fluoro-Jade B staining was obvious on digital imaging, the number of Fluoro-Jade B-positive cells per section was quantified with ImageJ (Analysis program developed at the National Institutes of Health (NIH), Bethesda, MD, USA). For each measurement, two blinded independent investigators counted 6–8 brains per group, 3 sections per brain.

### 4BAcetylcholinesterase Assay

The Amplex Red Acetylcholine/Acetylcholinesterase assay kit from Molecular probes Inc. (Invitrogen, Karlsruhe, Germany) was employed to estimate acetylcholinesterase activity using a fluorescence microplate reader. A working solution of 400 µM Amplex Red reagent containing 2 U/mL horseradish peroxidase, 0.2 U/mL choline oxidase and 100 µM acetylcholine [33] was prepared from the stock solutions. The reaction began when 100 µl of the working solution was added to each well containing the samples and controls diluted 20×. Samples and controls were tested in duplicates. Fluorescence emitted by the individual samples was measured in a fluorescence microplate reader (Infinite® 200, Tecan, Crailsheim, Germany) at an excitation of 560 nm and emission detection at 590 nm. Background fluorescence was eliminated by subtracting values derived from the negative control. Using the standard curve, concentrations of acetylcholinesterase from the samples of different groups were calculated.

### Statistical Evaluation

Experiments were performed in eight animals per group (n = 8). Values are presented as means ± standard error of the mean (SEM). Group effects were assessed by ANOVA followed by post hoc independent-sample t-test multiple comparison. p values are presented after Bonferroni correction. P<0.05 was considered as statistically significant.

## Results

### Surgery Combined with LPS**-**treatment is Associated with Increased IL-1beta Expression in the Cortex and Hippocampus which is Reduced by Physostigmine and Neostigmine

**Figure 1:**
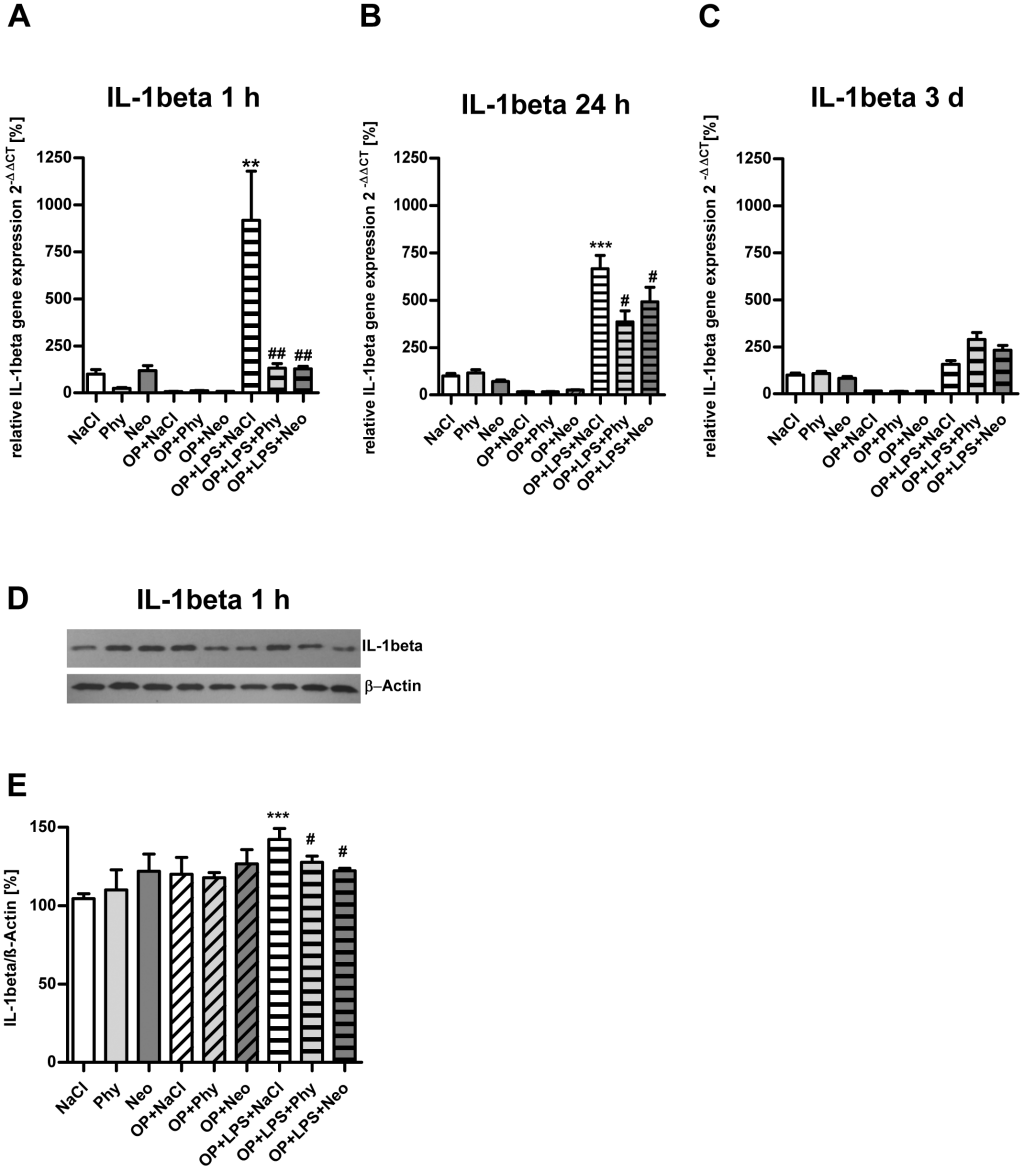
Physostigmine and neostigmine reduce surgery combined with LPS-induced *IL*-1beta expression in the cortex. IL-1beta expression was measured by quantitative Real-Time PCR in cortex samples extracted 1, 24, and 72 h postintervention (A–C). Also, IL-1beta expression was detected by western blot analysis in cortex samples extracted 1 h postintervention (D–E). Surgery combined with LPS-treatment resulted in an increased gene expression of *IL-1beta* after 1 and 24 h, which was decreased by physostigmine and neostigmine administration. Results of Real-Time PCR quantification are shown as mean ± SEM (n = 8 per group). Data are normalized to levels of saline treated rats (Control = 100%; bars represent mean ± SEM, n = 8 per group). Blots are representative of a series of three blots. The densitometric data represent the ratio of the IL-1beta band to the corresponding β-actin band density. ***P<0.001 and **P<0.01 represent the difference between LPS and saline treated groups. ^##^P<0.01 and ^#^P<0.05 represent the difference between LPS and physostigmine or neostigmine treated groups.

**Figure 2:**
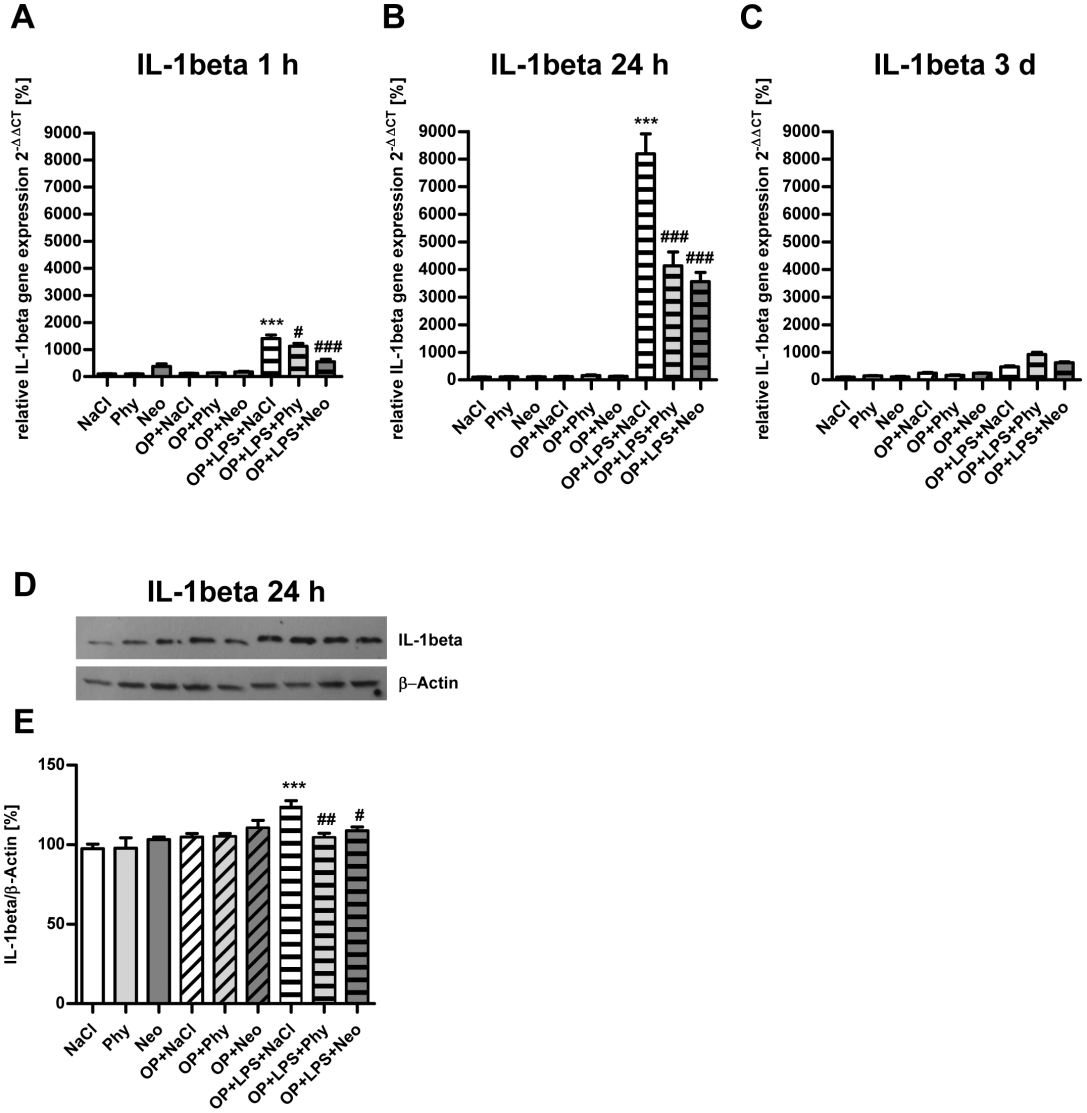
Physostigmine and neostigmine reduce surgery combined with LPS-induced IL-1beta expression in the hippocampus. IL-1beta expression was measured by quantitative Real-Time PCR in hippocampal samples extracted 1, 24, and 72 h postintervention (A–C). Additionally, IL-1beta expression was quantified by western blot analysis in hippocampal samples extracted 24 h postintervention (D–E). Surgery combined with LPS-treatment resulted in an increased expression of IL-1beta after 1 and 24 h and was reduced by physostigmine and neostigmine administration. Results of Real-Time PCR quantification are shown as mean ± SEM (n = 8 per group). Data are normalized to levels of saline treated rats (Control = 100%; bars represent mean ± SEM, n = 8 per group). Blots are representative of a series of three blots. The densitometric data represent the ratio of the IL-1beta band to the corresponding β-actin band density. ***P<0.001 represents the difference between LPS and saline treated groups. ^###^P<0.001, ^##^P<0.01, ^#^P<0.05 represent the difference between LPS and physostigmine or neostigmine treated groups.

As shown in Fig. 1, surgery combined with LPS-treatment led to increase *IL-1beta* gene expression in the cortex after 1 h (Fig. 1A) and 24 h (Fig. 1B) and was significantly reduced by physostigmine and neostigmine application. After 3 days (Fig. 1C) and 7 days (data not shown) no comparable expression of *IL-1beta* was detectable. Furthermore, the upregulation of IL-1beta in the cortex was confirmed after 1 h at the protein level by western blot analysis (Fig. 1D–E). Surgery in combination with LPS-treatment caused elevated *IL-1beta* gene expression in the cortex. The cholinergic activation by physostigmine and neostigmine minimized levels of IL-1beta protein expression. Similar results are shown for the hippocampus (Fig. 2). Surgery and treatment with LPS enhanced *IL-1beta* gene expression after 1 h (Fig. 2A) and 24 h (Fig. 2B) and was significantly reduced by physostigmine and neostigmine. After 3 days (Fig. 2C) and 7 days (data not shown) no comparable expression of *IL-1beta* was detectable. Western blot analysis in samples from the hippocampus extracted 24 h postintervention confirmed the upregulation of IL-1beta protein expression after surgery combined with LPS-treatment and its inhibition by physostigmine and neostigmine (Fig. 2D–E). No changes in the expression of the cytokines TNF-alpha and IL-10 were detected in the cortex or in the hippocampus (data not shown).

### Surgery Combined with LPS**-**treatment is Associated with Increased Gene Expression of Pro-inflammatory Cytokines in the Spleen which are Reduced by Physostigmine and Neostigmine

**Figure 3:**
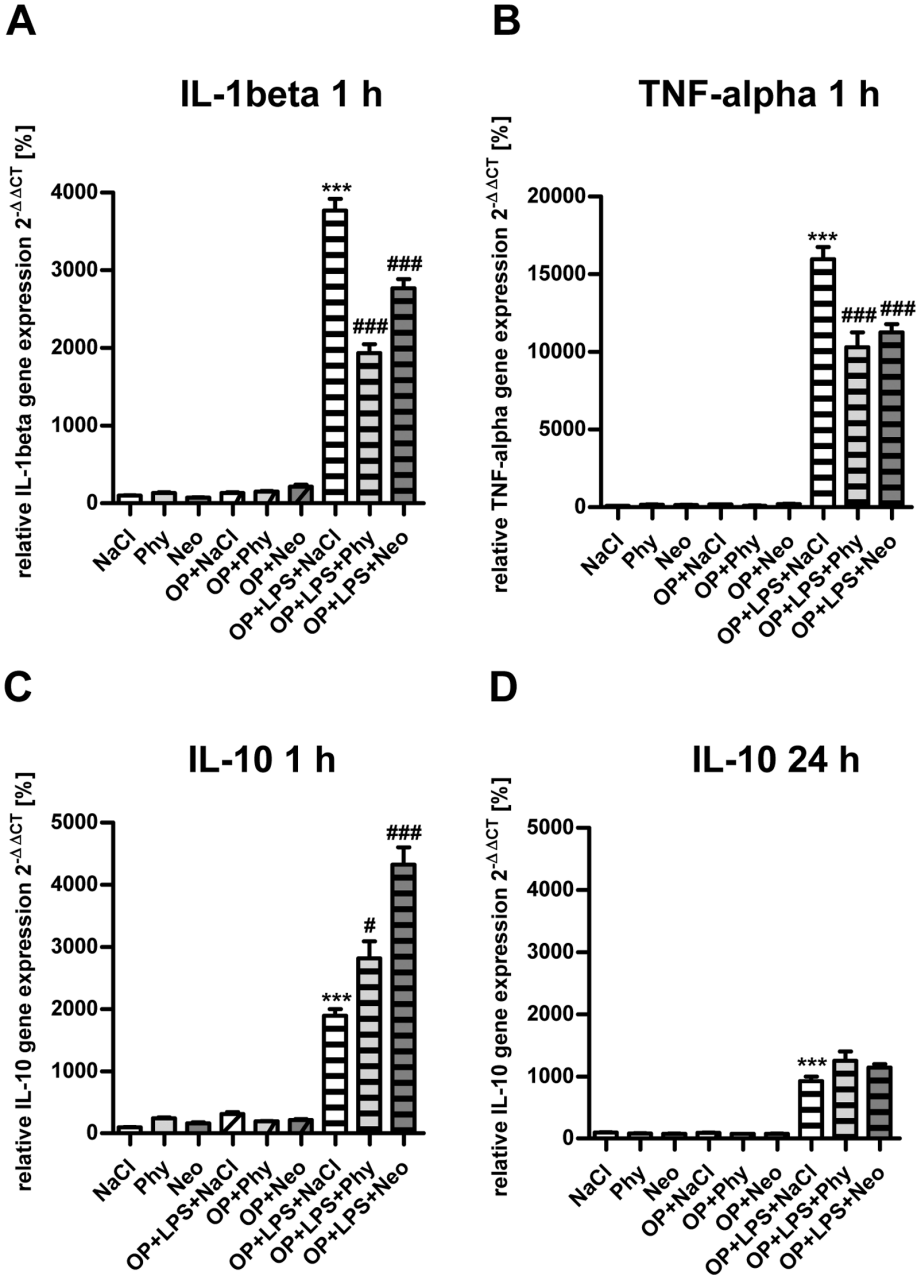
Physostigmine and neostigmine reduce surgery combined with LPS-induced *IL-1beta* and *TNF-alpha* gene expression in the spleen. *IL-1beta* (A) and *TNF-alpha* (B) expression was measured by quantitative Real-Time PCR in spleen samples extracted 1 h postintervention. Surgery combined with LPS-treatment resulted in an increased gene expression of *IL-1beta* and *TNF-alpha* after 1 h and was reduced by physostigmine and neostigmine treatment. *IL-10* expression was measured by quantitative Real-Time PCR in spleen samples extracted 1 h (C) and 24 h (D) postintervention. Surgery in combination with LPS-treatment led to an increased gene expression of *IL-10* after 1 and 24 h and is enhanced by physostigmine and neostigmine application after 1 h. Results of Real-Time PCR quantification are shown as mean ± SEM and normalized to levels of saline treated rats (Control = 100%, n = 8 per group). ***P<0.001 represents the difference between LPS and saline treated groups. ^###^P<0.001, ^#^P<0.05 represents the difference between LPS and physostigmine or neostigmine treated groups.

As shown in Fig. 3, surgery accompanied by LPS-treatment augmented *IL-1beta* (Fig. 3A) and *TNF-alpha* (Fig. 3B) gene expression in the spleen after 1 h. The cholinergic activation by physostigmine and neostigmine lowered levels of *IL-1beta* and *TNF-alpha* in the spleen. In comparison, after 24 h, 3 days and 7 days (data not shown) no increase of *IL-1beta* or *TNF-alpha* was detectable.

The expression of the anti-inflammatory gene *IL-10* was upregulated after 1 h (Fig. 3C) and still strong after 24 h, however less pronounced (Fig. 3D) following surgery combined with LPS-treatment. In contrast to the downregulation of the pro-inflammatory cytokines, physostigmine and neostigmine significantly enhanced the gene expression of *IL-10* in the spleen but only after 1 h. Both AChE inhibitors have no effect on the expression of *IL-10* after 24 hours (Fig. 3D). After 3 and 7 days (data not shown) no increase of *IL-10* was detectable.

### Surgery Combined with LPS-treatment is Associated with Increased Protein Levels of Pro-inflammatory Cytokines in the Spleen and Plasma which are Reduced by Physostigmine and Neostigmine

**Figure 4:**
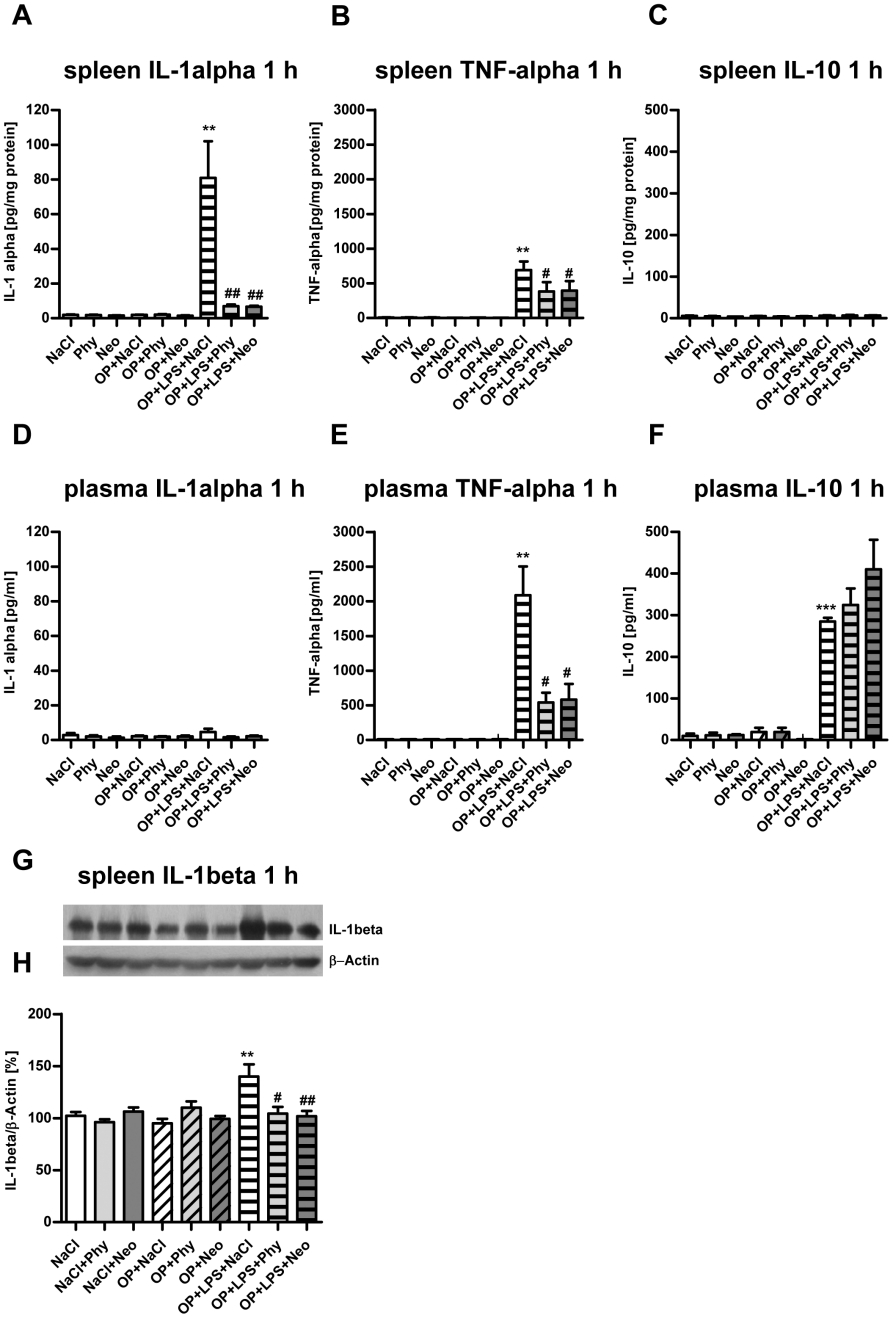
Physostigmine and neostigmine reduce surgery combined with LPS-induced IL-1beta and TNF-alpha protein expression in spleen and plasma. IL-1-alpha, TNF-alpha and IL-10 were measured by Cytometric bead array analysis in spleen (A–C) and plasma (D–F) samples 1 h postintervention. Surgery and LPS-treatment resulted in an increased protein expression of IL-1alpha and TNF-alpha in the spleen after 1 h. Treatment with the AChE inhibitors physostigmine and neostigmine significantly reduced the expression of IL-1alpha in the spleen. In plasma, surgery accompanied by LPS-treatment caused an increased protein expression of TNF-alpha and IL-10. TNF-alpha concentration was significantly diminished by physostigmine and neostigmine application, whereas the expression of IL-10 did not change significantly. Additionally, IL-1beta expression was quantified by western blot analysis in spleen samples extracted 1 h postintervention (G–H). Surgery and LPS-treatment resulted in an increased protein expression of IL-1beta in the spleen after 1 h. Treatment with the AChE inhibitors physostigmine and neostigmine significantly reduced the expression of IL-1beta in the spleen. Results of Cytometric bead array and western blot analysis are shown as mean ± SEM (n = 8 per group). ***P<0.001, **P<0.01 represent the difference between LPS and saline treated groups. ^#^P<0.05, ^##^P<0.01 represent the difference between LPS and physostigmine or neostigmine treated groups.

Cytometric bead array analysis in samples from the spleen extracted 1 h postintervention confirmed the upregulation of pro-inflammatory cytokines IL-1alpha (Fig. 4A) and TNF-alpha protein expression after 1 h (Fig. 4B) as well as the inhibition of IL-1alpha by physostigmine and neostigmine. No upregulation of IL-10 protein expression 1 h following surgery treatment with LPS was detectable in the spleen (Fig. 4C). As shown in Fig. 4D, surgery accompanied by LPS-treatment did not trigger an increased IL-1alpha protein expression in the plasma. However, TNF-alpha protein expression was elevated after 1 h (Fig. 4E). The enhanced expression of TNF-alpha after 1 h was significantly decreased by physostigmine and neostigmine. Following surgery and LPS-treatment, IL-10 protein expression in the plasma was detectable after 1 h postintervention and did not change significantly using physostigmine and neostigmine (Fig. 4F). After 24, 72 h or 7 days no increase of pro- or anti-inflammatory protein expression was detectable (data not shown). Additionally, IL-1beta expression was quantified by western blot analysis in spleen samples extracted 1 h postintervention (Fig. 4G–H). Surgery and LPS-treatment resulted in an increased protein expression of IL-1beta in the spleen after 1 h. Treatment with the AChE inhibitors physostigmine and neostigmine significantly reduced the expression of IL-1beta in the spleen.

### Surgery Combined with LPS**-**treatment is Associated with Increased Neurodegeneration and Activity of Acetylcholinesterase in the Cortex and Hippocampus which are Reduced by Physostigmine and Neostigmine

**Figure 5:**
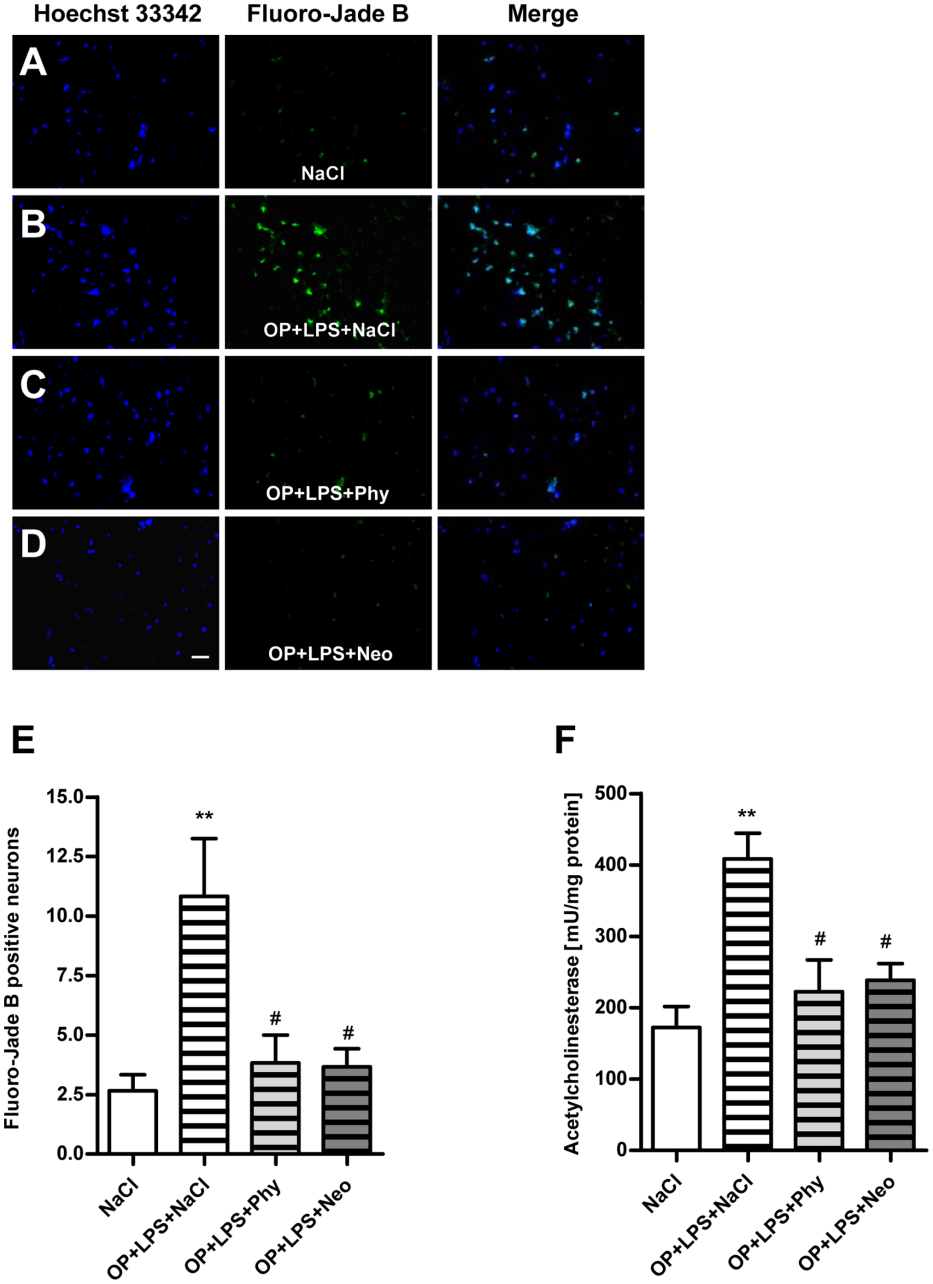
Physostigmine and neostigmine reduce surgery combined with LPS-induced neurodegeneration and activity of acetylcholinesterase in the cortex. Representative photomicrographs (original magnification X200) of Hoechst 33342 (left panel) and Fluoro-Jade B (right panel) staining from the cortex (Scale bar = 20 µm) of adult rats which were treated with saline (A), surgery combined with LPS-treatment+saline (B), surgery combined with LPS-treatment+physostigmine (C) or surgery combined with LPS-treatment+neostigmine (D). (E) Comparison of the number of Fluoro-Jade B-positive cells from the cortex of adult rats. (F) Activity of acetylcholinesterase in the cortex after 1 h. Surgery combined with LPS-treatment triggered the number of degenerated neurons and the activity of acetylcholinesterase in the cortex after 1 h. Physostigmine and neostigmine led to significantly reduced neuronal damage and activity of acetylcholinesterase. Number of Fluoro-Jade B-positive cells are shown as mean ± SEM (n = 5–6 per group). **P<0.01 represents the difference between surgery combined with LPS-treatment and saline treated groups. ^#^P<0.05 represents the difference between surgery together with LPS-treatment and in combination with physostigmine or neostigmine treated groups.

To examine if neuroinflammation is associated with neurodegeneration, brain sections were stained with Fluoro-Jade B, a marker for neurons that undergo cell death and additionally with Hoechst 33342, a marker for DNA. Compared to the control group (Fig. 5A) surgery in combination with LPS-treatment resulted in an increased number of neuronal damage in the cortex after 24 h (Fig. 5B). Treatment with physostigmine (Fig. 5C) and neostigmine (Fig. 5D) led to significantly reduced neuronal damage. The comparison of the number of Fluoro-Jade B-positive cells in the cortex is shown in Fig. 5E. Compared to the control group surgery together with LPS-treatment caused an increased activity of acetylcholinesterase in the cortex after 24 h, which could be diminished by physostigmine and neostigmine (Fig. 5F).

**Figure 6:**
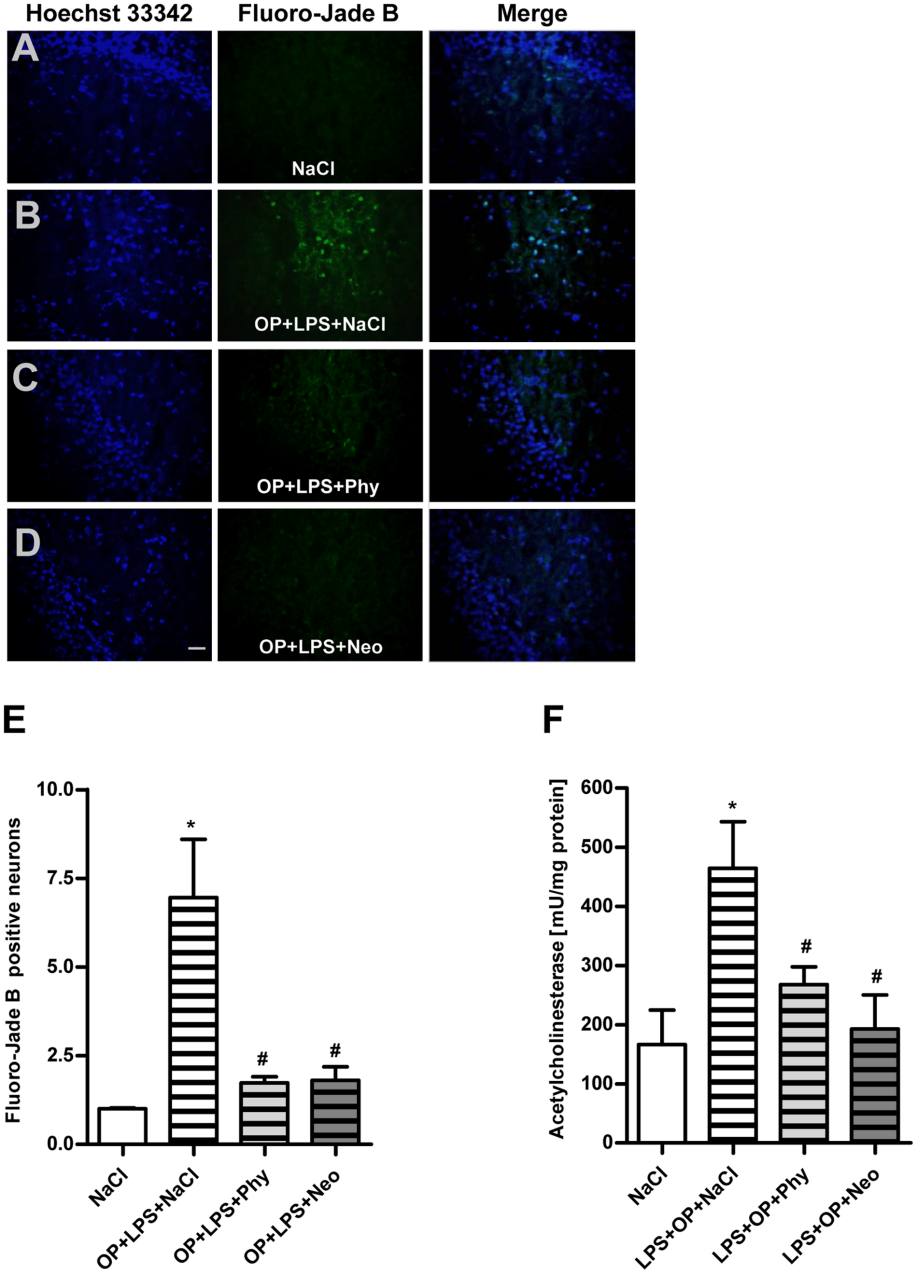
Physostigmine and neostigmine reduce surgery combined with LPS-induced neurodegeneration and activity of acetylcholinesterase in the hippocampus. Representative photomicrographs (original magnification X200) of Hoechst 33342 (left panel) and Fluoro-Jade B (right panel) staining from the hippocampal dentate gyrus granule cell layer (Scale bar = 20 µm) of adult rats which were treated with saline (A), surgery combined with LPS-treatment+saline (B), surgery combined with LPS-treatment+physostigmine (C) or surgery combined with LPS-treatment+neostigmine (D). (E) Comparison of the number of Fluoro-Jade B-positive cells from the hippocampal dentate gyrus granule cell layer of adult rats. (F) Activity of acetylcholinesterase in the hippocampus after 24 h. Surgery combined with LPS-treatment triggered the number of degenerated neurons and the activity of acetylcholinesterase in the hippocampus after 24 h. Physostigmine and neostigmine led to significantly reduced neuronal damage and activity of acetylcholinesterase. Number of Fluoro-Jade B-positive cells are shown as mean ± SEM (n = 5–6 per group). *P<0.05 represents the difference between surgery combined with LPS-treatment and saline treated groups. ^#^P<0.05 represents the difference between surgery together with LPS-treatment and in combination with physostigmine or neostigmine treated groups.

Compared to the control group (Fig. 6A) surgery in combination with LPS-treatment resulted in an increased number of neuronal damage after 24 h (Fig. 6B) in the dentate gyrus of the hippocampus. Treatment with physostigmine (Fig. 6C) and neostigmine (Fig. 6D) led to significantly reduced neuronal damage. The comparison of the number of Fluoro-Jade B-positive cells from the dentate gyrus granule cell layer of the hippocampus is shown in Fig. 6E. Compared to the control group surgery together with LPS-treatment caused an increased activity of acetylcholinesterase in the hippocampus after 24 h, which could be diminished by physostigmine and neostigmine (Fig. 6F).

## Discussion

The study at hand could show that surgery combined with LPS-treatment leads to a significant upregulation of the pro-inflammatory cytokine IL-1beta in the cortex and hippocampus after 1 and 24 h. These findings are in line with several studies, which report that the production of IL-1beta in the brain in general and in the hippocampus in particular is induced by LPS [35], [36]. Figaldo et al. reported that sub-clinical infection may sensitise the immune system augmenting the severity of post-operative cognitive dysfunction [3].

IL-1beta mediates an inflammatory process in the hippocampus that underlies memory impairment [37]. IL-1 is a cytokine, produced mainly by monocytes and macrophages, as well as by glia cells and neurons within the brain. Various studies have shown that IL-1beta plays a potential role in the pathophysiology of neurodegenerative diseases [38]. One consequence of an increased IL-1beta concentration is the inhibitory effect on long-term potentiation in dentate gyrus of the hippocampus [39], [40]. Long-term potentiation is widely considered one of the major cellular mechanisms that underlie learning and memory [41], [42].

The present data show, that in our model of adult rats (3 months) surgery (laparotomy) alone does not induce a neuroinflammatory response. These findings confirm the studies done by Barrrientos et al., showing an increase in IL-1beta, which correlates with hippocampal-dependent memory impairment in old rats (24 months) after laparotomy but not in adult rats (3 months). The authors demonstrated that aged animals are more vulnerable to cognitive decline after a peripheral immune challenge [43]. The expression of IL-1beta and IL-1 type 1 receptors is particularly high in the hippocampus. The concentration of IL-1beta is elevated in the hippocampus of old rats [44]. The aging brain seems to be more susceptive to peripheral bacterial infection and surgical stress and reacts with specifically exaggerated and persistent IL-1beta response [41], [45]. This could be a reason why especially elderly patients suffer from POCD [8], [9], [46]–[48].

Cibelli and colleagues demonstrated that a peripheral orthopedic surgery-induced innate immune response triggers an IL-1beta-mediated inflammatory process in the hippocampus underlying memory impairment [49]. Therefore, we concluded that laparotomy alone is not sufficient to induce IL-1beta in the brain of adult rats. However, in combination with LPS, it serves as a model for neuroinflammation to examine the effectiveness of cholinergic stimulation.

The result of this study demonstrates that the raise of IL-1beta in the hippocampus and cortex was significantly reduced by intraperitoneal administration of physostigmine and neostigmine, before surgery following LPS-treatment. It has been shown earlier that the administration of CNS-active acetylcholinesterase inhibitors depletes systemic pro-inflammatory cytokines and ameliorates both central and peripheral inflammation [50], [51].

In contrast to the centrally-acting acetylcholinesterase inhibitor physostigmine, neostigmine does not cross the blood-brain barrier, and thus has no effect on AChE within the brain. Interestingly, in our study neostigmine inhibited the production of IL-1beta within the cortex and hippocampus. It could be demonstrated recently, that neostigmine has no anti-inflammatory effect in neuroinflammation induced by an intracerebroventricular injection of LPS [52]. A possible explanation for our results would be the reduction of the peripheral expression of pro-inflammatory cytokines like IL-1beta and TNF-alpha, which decreases the subsequent signal of the vagus nerve to the brain and leads to a reduced neuroinflammation. This confirmed the possibility to control postoperative neuroinflammation in the brain by suppressing the peripheral immune response during surgical treatment. We suspect that neostigmine has not the same effect in patients with decreased levels of cholinergic neurotransmission or increased expression of IL-1beta in the brain that has been related to progressing memory deficits with aging. Recently, it has been demonstrated that a single intracisternal but not peripheral administration of an interleukin-1 receptor antagonist throughout surgery was sufficient to block both the neuroinflammatory response and the behavioral deficit [43]. Therefore, we deduce that the peripheral downregulation of the IL-1beta pathway is not sufficient since other proinflammatory cytokines like TNF-alpha send signals from the vagus nerve to the brain.

To verify the peripheral cytokine expression after surgery alone or in combination with LPS-treatment, pro- and anti-inflammatory cytokines were measured in plasma and spleen. As might have been expected, surgery combined with LPS-treatment led to a significant upregulation of the pro-inflammatory cytokines IL-1beta, TNF-alpha as well as the anti-inflammatory cytokine IL-10 in spleen and plasma. In the present study it was observed that the gene expression of the pro-inflammatory cytokines *IL-1beta* and *TNF-alpha* increased in spleen tissue 1 h after surgery and LPS-treatment. The protein IL-1alpha remains in the spleen as it could not be detected in plasma, while protein expression of TNF-alpha was mainly detected in plasma. Physostigmine and neostigmine significantly decreased the gene expression of *IL-1beta* and *TNF-alpha* as well as the protein expression of IL-1 alpha and TNF-alpha. Unfortunately, the measurement of IL-1beta protein expression was not available for the BD™ CBA Flex Set, so instead IL-1alpha was determined. To see if IL-1beta is similarly regulated in the spleen we additionally quantified IL-1beta expression by western blot analysis in spleen samples extracted 1 h postintervention. Surgery and LPS-treatment resulted in an increased protein expression of IL-1beta in the spleen after 1 h. Treatment with the AChE inhibitors physostigmine and neostigmine significantly reduced the expression of IL-1beta in the spleen.

The expression of the anti-inflammatory gene *IL-10* in the spleen was upregulated after 1 h and to a lesser extent after 24 h following surgery together with LPS-treatment. In contrast to the downregulation of the pro-inflammatory cytokines, physostigmine and neostigmine significantly but only briefly augmented the gene expression of *IL-10* in spleen tissue after 1 h. The protein expression of IL-10 was detectable in plasma, and here, physostigmine and neostigmine did not alter the expression of IL-10 significantly. Our data are consistent with the results of Tracey et al. [15]. They describe the *inflammatory reflex* that controls the innate immune responses by a mechanism that targets pro-inflammatory cytokines but do not alter the production of the anti-inflammatory cytokine IL*-*10. IL-10 signaling suppresses nuclear localization and activation of NF-kB, leading to the inhibition of pro-inflammatory cytokine gene transcription in human monocytes [53]. Li and colleagues reported that IL-10 upregulates the expression of α7 nAChR in RAW264.7 cells [54]. Furthermore, Lynch et al. reported that LPS leads to an increased expression of IL-beta and cell death in the hippocampus, which can be reduced by IL-10 [55].

In order to demonstrate whether the enhanced level of IL-1beta induces cell death in the cortex and hippocampus, we stained brain sections with Fluoro-Jade B. It has been proposed that the impairment in long-term potentiation, the cellular mechanism that underlies learning and memory, is a consequence of the increase in IL-1beta concentration and IL-1beta-induced cell death [56]. Our results demonstrated that IL-1beta is associated with enlarged neurodegeneration in the cortex and the dentate gyrus of the hippocampus and this neurodegeneration is minimized by the AChE inhibitors physostigmine and neostigmine. Furthermore, it could be shown that the activity of AChE is enhanced after surgery in combination with LPS and diminished after treatment with physostigmine and neostigmine. Our finding is supported by other groups, who found increased number of cell deaths that strongly correlated to AChE activity [31], [52], [57], [58]. Interestingly, even neostigmine inhibits the activity of acetylcholinesterase in the hippocampus although it cannot pass the blood-brain barrier, thus having no effect of AChE within the brain. This confirms once again that neostigmine acts indirectly on the cortex and hippocampus by peripherally reducing the expression of pro-inflammatory cytokines. The results provide evidence for the relationship between the immune system and the CNS and suggest a link between IL-1beta, AChE activity and dysfunction in the cortex and hippocampus. Therefore, our finding is of important clinical value. The stimulation of the cholinergic anti-inflammatory pathway through AChE inhibitors can reduce the production of IL-1beta in the brain which in turn protects against neurodegeneration.

### Conclusion

Our study showed for the first time, that physostigmine and neostigmine can decrease the expression of IL-1beta and cortical/hippocampal neurodegeneration after surgery and subsequent LPS-treatment. Since neuroinflammation is involved in brain dysfunction, cholinergic activation seems to be a potential neuroprotective treatment.

### Limitations

Further studies are required whose aim should be the examination of memory impairment in this animal model. There is compelling evidence that relative expression of IL-1beta is a key element in the development of postoperative cognitive dysfunction. IL-1beta is mediating inflammation and causing hippocampal-dependent memory impairment [37], [49], [59]. More investigations are necessary to learn about the protective effect of cholinergic stimulation and its cellular and molecular mechanisms.
