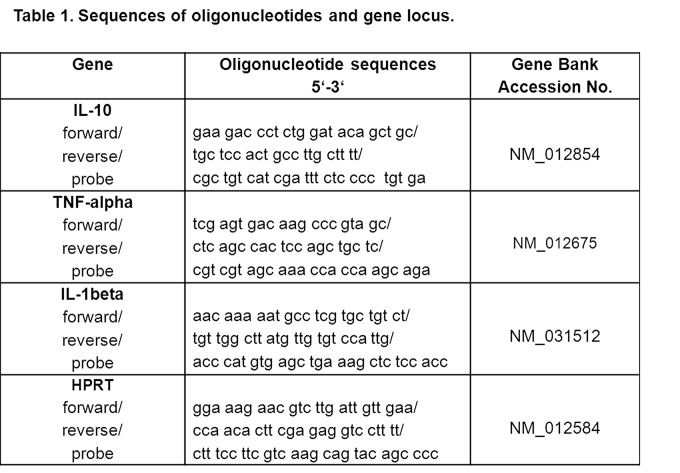# Correction: Acetylcholinesterase Inhibitors Reduce Neuroinflammation and -Degeneration in the Cortex and Hippocampus of a Surgery Stress Rat Model

**DOI:** 10.1371/annotation/a4f6882e-e174-4bfb-bbcc-8d380d5dd225

**Published:** 2013-11-06

**Authors:** Alexander Kalb, Clarissa von Haefen, Marco Sifringer, Annalena Tegethoff, Nadine Paeschke, Mariya Kostova, Aarne Feldheiser, Claudia D. Spies

In Table 1, the labels forward, reverse, and probe were not distinguished as separate rows. Please see the corrected Table 1 here: 

**Figure:**